# Integrated strategies for type 2 diabetes prevention: The role of diet and exercise

**DOI:** 10.3934/publichealth.2025024

**Published:** 2025-04-08

**Authors:** Nicola Tecce, Mattia Proganò, Davide Menafra, Annamaria Docimo, Stefano Zarrilli, Roberta Scairati, Anna Lisa Pelosi, Rosario Pivonello, Annamaria Colao

**Affiliations:** 1 Department of Clinical Medicine and Surgery, Department of Endocrinology, University Federico II of Naples, Naples, Italy; 2 Unesco Chair for Health Education and Sustainable Development, Federico II University, Naples, Italy

**Keywords:** type 2 diabetes, dietary patterns, exercise regimens, glycemic control, prevention strategies

## Abstract

**Background:**

Type 2 diabetes (T2D) is a prevalent global health problem largely caused by lifestyle factors, including poor diet and physical inactivity. With the increasing incidence of T2D, effective prevention strategies are urgently needed. This review examines the role of different dietary patterns and exercise regimens in the prevention and management of T2D, focusing on their effects on glycemic control, insulin sensitivity, and cardiovascular health.

**Methods:**

A narrative review was conducted synthesizing evidence from clinical trials, cohort studies, and meta-analyses. The review focused on the effects of low-carbohydrate diets (LCDs), Mediterranean diets (MDs), plant-based diets, high-protein diets (HPDs), and various exercise regimens, including aerobic, resistance, and high-intensity interval training (HIIT).

**Results:**

Dietary interventions, particularly MD and plant-based diets, are associated with improved glycemic control and reduced risk of developing T2D. LCDs show short-term benefits for weight management and HbA1c reduction, although adherence remains a challenge. HPDs show mixed results, with some benefits for lipid profiles but inconsistent effects on glycemic control. Exercise programs, especially combined aerobic and resistance training, significantly improve glycemic control, insulin sensitivity, and cardiovascular risk factors. HIIT emerges as an effective option for improving metabolic health in individuals at risk for or living with T2D.

**Conclusions:**

A combination of dietary modification and physical activity, particularly Mediterranean and plant-based diets coupled with combined aerobic and resistance exercise, appears to be the most effective strategy for the prevention and management of T2D. Future research should focus on personalized approaches that integrate both diet and exercise to tailor interventions to individual patient needs.

## 1. Introduction

Type 2 diabetes (T2D) is a chronic disease that affects the body's ability to regulate blood glucose levels. It is characterized by insulin resistance, which is the reduced responsiveness of cells to insulin, and a reduction in insulin production by the pancreas. This results in elevated blood sugar levels (hyperglycemia) [1]. T2D is a highly prevalent disease worldwide, affecting approximately 425 million adults. With steadily increasing incidence rates, it is one of the most significant public health problems of the 21^st^ century [2]. The International Diabetes Federation (IDF) projects that T2D will affect approximately 700 million individuals by 2045, representing a significant and alarming increase that underscores the urgent need for intensified prevention and treatment efforts [2].

### 1.1. Role of lifestyle in T2D prevalence

The elevated incidence and prevalence of T2D can be attributed to the detrimental impact of adverse lifestyle factors, including physical inactivity and an unhealthy diet, which contribute to the accumulation of adipose tissue and the development of obesity. These factors are closely associated with the rising prevalence of T2D [3]. Prospective studies have shown that increases in body weight, and therefore body mass index (BMI), over time are associated with a significantly increased risk of developing T2D [4]. A growing body of evidence indicates that an increase in BMI of 5 kg/m² is associated with a more than two-fold increase in the risk of death from T2D, from the upper limit of normal weight (25 kg/m²) to the lower limit of first-degree obesity (30 kg/m²) [5]. The metabolic alterations associated with adipose tissue accumulation encompass the augmented release of fatty acids into the circulatory system, diminished insulin-sensitive tissue responsiveness, and ectopic fat deposition, which may occur prior to the onset of glucose homeostasis irregularities [6]. In the initial stages of T2D, metabolic alterations associated with overweight and obesity, including prediabetes, result in gradual elevations in fasting blood glucose (FBG) and postprandial glucose [7]. This is frequently obscured by the pancreas's compensatory production of elevated insulin levels (hyperinsulinemia), which facilitates glycemic control for a period of time [7]. Nevertheless, as insulin resistance advances, the hypersecretory function of the pancreas is unable to meet the body's requirements, resulting in β-cell dysfunction, elevated glucose levels, and ultimately, a T2D diagnosis [8].

### 1.2. Visceral obesity and ectopic fat

It is important to note that the risk of developing T2D is not solely tied to obesity. Rather, it is also associated with visceral obesity, defined as the accumulation of fat around the abdominal organs and ectopic fat deposition (e.g., hepatic steatosis). These conditions are more predictive of T2D risk than BMI alone and thus should be considered in addition to BMI when assessing risk [9]. The available evidence indicates that individuals with low visceral and ectopic fat, despite being obese, may be free of metabolic complications [10],[11]. Conversely, some individuals develop T2D despite being of normal weight or slightly overweight. This is due to the accumulation of visceral fat, chronic inflammation, and a reduction in lean muscle mass, which can result from inadequate physical activity [12].

### 1.3. Clinical evidence on diet and exercise

A review of the literature over the past 20 years reveals a consistent finding: diet and exercise play a significant role in the prevention and management of T2D [13],[14]. A variety of dietary approaches have been subjected to investigation, including low-carbohydrate diets, the Mediterranean diet (MD), plant-based diets, and high-protein, low-fat regimens [15]. Each of these approaches offers distinctive advantages with respect to insulin sensitivity, glycemic control, and cardiovascular risk reduction. Similarly, evidence indicates that a combination of dietary modifications and physical activity is an efficient approach to reducing the risk of developing T2D by enhancing metabolic health [16]. The World Health Organization (WHO) recommends that individuals engage in at least 150–300 minutes per week of moderate-intensity aerobic exercise regimens or 75–150 minutes of vigorous-intensity aerobic physical activity, coupled with resistance training twice per week, to mitigate the risks associated with cardiovascular disease and T2D [17]. A number of exercise regimens have been subjected to investigation, including aerobic training, resistance exercise, HIIT, and combinations thereof [18]–[20].

### 1.4. Objectives of the review

The objective of this review is to synthesize the findings of clinical trials that have investigated the effects of different dietary patterns and exercise regimens on the development and management of T2D. The focus will be on the role of diet composition, including carbohydrate quality and restriction, and the impact of various exercise protocols. Finally, recommendations will be offered for patients, healthcare providers, and future research directions.

### 1.5. Methodology

A systematic literature search was conducted to identify relevant studies for this review. A total of 3263 articles were initially retrieved from PubMed, Medline (via Web of Science), Cochrane Library, ProQuest, Scopus, and Medline Ovid. The search employed a combination of keywords and MeSH terms such as “type 2 diabetes”, “diet”, “exercise”, “insulin resistance”, “weight loss”, “lifestyle intervention”, and “metabolic syndrome”, with Boolean operators (AND, OR) used to maximize scope.

**Figure 1.**
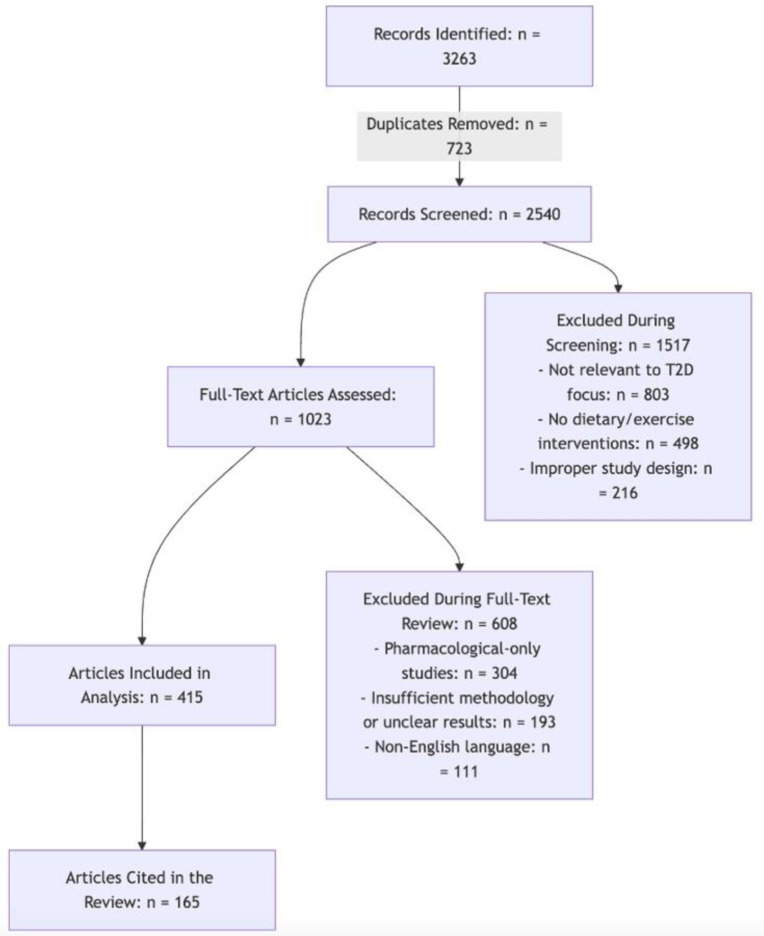
Flowchart depicting the systematic selection process for included studies.

The articles underwent a rigorous multi-step screening process (Figure 1):

1. Title and abstract screening: After removing duplicates (723 articles), 2540 articles remained. Of these, 1517 articles were excluded for the following reasons: not focused on T2D prevention or management (803 articles); lack of dietary or exercise intervention (498 articles); study design not meeting inclusion criteria, such as case reports or editorials (216 articles).

2. Full-text review: The remaining 1023 articles underwent full-text assessment. During this stage, 608 articles were excluded for the following reasons: irrelevant outcomes, such as pharmacological-only interventions (304 articles); insufficient methodological detail or unclear results (193 articles), non-English language (111 articles).

3. Final inclusion: A total of 415 articles met all inclusion criteria and were included in the review. These studies consisted of clinical trials, cohort studies, and meta-analyses examining dietary patterns, exercise regimens, or their combined effects on glycemic control, insulin sensitivity, and cardiovascular health.

4. To assess the quality of the included studies, we prioritized randomized controlled trials (RCTs), cohort studies, and meta-analyses, applying elements of the Cochrane Risk of Bias (RoB 2.0) tool and Newcastle–Ottawa Scale (NOS) for rigor assessment. Studies with adequate sample sizes, robust statistical analyses, and clear confidence intervals were favored, along with recent publications from high-impact, peer-reviewed journals. Of the 415 eligible articles, 165 were selected based on methodological strength, clinical relevance, and direct focus on diet and/or exercise in T2D prevention and management.

This detailed selection process ensures a robust and representative synthesis of evidence for the review.

### 1.6. Novel contributions of this review

This review offers a novel synthesis of current evidence by bridging the gap between dietary and exercise interventions, emphasizing their combined effects on glycemic control, insulin sensitivity, and cardiovascular health. It uniquely highlights the role of personalized strategies tailored to individual metabolic profiles, dietary preferences, and exercise feasibility, while integrating emerging insights on the gut microbiota's contribution to T2D. By focusing on the synergistic potential of combined interventions and advocating for a patient-centric approach, the review provides actionable recommendations and sets a foundation for future research in T2D prevention and management.

## 2. Impact of different diets on type 2 diabetes

### 2.1. Low-carbohydrate diets (LCDs)

#### 2.1.1. Mechanisms of action

LCDs restrict carbohydrate consumption to a daily intake of less than 130 g, with an increased focus on protein and fat intake [21]. In very low–calorie ketogenic diets (VLCKD), carbohydrate intake may be restricted to a minimal amount, typically between 20–50 g per day [22]. This represents a notable decrease in comparison to the World Health Organization (WHO) recommended daily carbohydrate intake of 45%–65% of calories for healthy adults [23]. The reduction in carbohydrate intake that is characteristic of these diets results in the depletion of glycogen stored within the liver and muscles, which in turn leads to a reduction in blood glucose levels [24]. In response, the body increases fat oxidation and enters a state of ketosis, whereby fatty acids are converted into ketone bodies to meet energy demands [24]. Additionally, reduced insulin levels lead to decreased fat storage and improved insulin sensitivity, which helps in weight management and glycemic control [25],[26]. Gluconeogenesis employs substrates such as lactic acid, glycerol, and amino acids (e.g., alanine and glutamine) for the production of glucose [27]. Conversely, ketogenesis involves the conversion of fatty acids released from adipose tissue into ketone bodies (acetoacetate, β-hydroxybutyrate, and acetone) [27]. In contrast to fasting states, LCDs provide exogenous protein and fat to meet energy needs, thereby reducing the loss of lean body mass (LBM) and helping to maintain stable glucose levels [28]. This alteration in metabolic processes, in conjunction with diminished insulin secretion, impedes the accumulation of adipose tissue (adipogenesis), thereby facilitating weight reduction and enhancing glycemic control [29].

#### 2.1.2. Clinical evidence for glycemic control and weight management

A number of studies have demonstrated the beneficial effects of LCDs on glycemic control and weight management in individuals with T2D [26]. A randomized controlled trial (RCT) revealed that participants on an LCD exhibited significantly greater reductions in HbA1c after six months in comparison to those following a standard diet (−0.23%; 95% CI: −0.32% to −0.14%; p < 0.001) [30]. Additionally, there were notable decreases in fasting blood glucose levels (−10.3 mg/dL; 95% CI: −15.6 to −4.9 mg/dL; p < 0.001) and body weight (−5.9 kg; 95% CI: −7.4 to −4.4 kg) [30]. However, the study did not evaluate whether these benefits were independent of weight loss. In other studies, LCDs have been shown to result in significant improvements in glycemic control, even in the absence of weight loss. For example, a study of participants with T2D who were following a eucaloric diet (i.e., a diet that did not result in weight loss) found that reducing dietary carbohydrates to 31% of total energy intake led to a significant decrease in postprandial glucose and insulin response after just 48 h [31]. Similarly, another six-week study involving obese participants with T2D found that a carbohydrate-restricted diet resulted in significant reductions in fasting blood glucose (p < 0.05), HbA1c levels (p < 0.001), and ectopic fat content in the liver (p < 0.01) and pancreas (p < 0.05) [32]. A meta-analysis corroborated these findings, demonstrating that LCDs were more efficacious than high-carbohydrate diets in reducing HbA1c levels by approximately 0.34 percentage points or 3.7 mmol/mol after a period of 3–6 months (p = 0.02) [33].

#### 2.1.3. Challenges and long-term considerations

While LCDs show clear short-term benefits in improving glycemic control and promoting weight loss, their long-term effectiveness is less certain due to challenges with adherence. Many individuals find it difficult to maintain low-carbohydrate eating habits, leading to a reduction in dietary compliance over time, particularly after one year [35]. Additionally, although LCDs can lower triglycerides and increase HDL cholesterol, there are concerns regarding the potential impact of increased dietary fat (especially saturated fats) on LDL cholesterol levels and cardiovascular risk [35]. Long-term studies are necessary to fully understand the cardiovascular effects of LCDs and whether these diets can be safely recommended for extended periods.

In conclusion, LCDs provide significant benefits in the short term by improving insulin sensitivity, glycemic control, and weight management in individuals with or at risk of T2D. However, the sustainability of these diets and their long-term impact on cardiovascular health require further exploration.

### 2.2. Mediterranean diet

#### 2.2.1. Key components and nutritional profile

The MD is widely recognized for its health benefits and is characterized by a high intake of fruits, vegetables, legumes, whole grains, nuts, and olive oil, with moderate consumption of fish, dairy, and red wine, and a limited intake of red meat and sweets [34]. This dietary pattern reflects the traditional eating habits of Mediterranean countries and has been extensively studied for its role in reducing the risk of cardiovascular diseases, improving metabolic health, and preventing T2D [35]. In light of the aforementioned considerations, the Fifth Session of the Intergovernmental Committee of UNESCO resolved in 2010 to include the MD in the Representative List of the Intangible Cultural Heritage of Humanity [36]. The consumption of fiber from fruits, vegetables, and whole grains has been demonstrated to facilitate the slowing of carbohydrate absorption, thereby moderating postprandial blood glucose spikes [37].

#### 2.2.2. Evidence from clinical trials

The MD has emerged as a powerful tool in managing and preventing both obesity and T2D, demonstrating consistent benefits for glycemic control, insulin sensitivity, and weight management. Studies highlight the MD's unique composition—rich in monounsaturated fats from olive oil, fiber from fruits, vegetables, and legumes, and polyphenols—which collectively reduce systemic inflammation and improve metabolic parameters [38],[39]. The PREDIMED study provided robust evidence linking the MD to a 52% reduced risk of developing T2D while also reversing metabolic syndrome in 13.7% of participants [40]. Additionally, the MD supports sustainable weight loss and improved cardiovascular outcomes, essential in addressing *diabesity*, the intersection of obesity and diabetes [39],[41]. The effectiveness of the MD in reducing the risk of developing T2D has been demonstrated by numerous prospective studies. The results of a meta-analysis of eight studies revealed that individuals who adhere closely to the MD are 13% less likely to develop T2D than individuals with lower adherence levels [37]. The ATTICA study, which included over 3000 participants, demonstrated that greater adherence to the MD was associated with improved fasting glucose levels, lower insulin concentrations, and a superior Homeostatic Model Assessment (HOMA) index, particularly in women [42]. Another notable study revealed that individuals who adhered to the MD diet exhibited a 49% reduction in the 10-year incidence of T2D in men and 69% in women, further underscoring its long-term protective effects [43]. The MD is also associated with lower levels of oxidative stress, reduced systemic inflammation, and improved gut microbiota, all of which contribute to its ability to mitigate metabolic risk factors for T2D [44].

#### 2.2.3. Challenges and long-term considerations

Despite the numerous benefits, the MD does present certain challenges. One potential limitation is that the MD may not result in immediate weight loss in comparison to more restrictive diets, such as LCDs. Furthermore, some individuals may perceive the MD to be costlier or less accessible due to its emphasis on fresh produce, fish, and olive oil, which may be financially burdensome for specific demographic groups. Another potential challenge is that while the MD is sustainable for long-term adherence due to its variety and palatability, it may require significant behavioral changes for individuals who are accustomed to highly processed or fast food–heavy diets. Nevertheless, the MD's versatility and adaptability to different cultural contexts make it an optimal dietary recommendation for both T2D prevention and overall metabolic health.

### 2.3. Plant-based diets

#### 2.3.1. Variations (vegan, vegetarian, flexitarian) and nutritional focus

Plant-based diets are centered around the consumption of fruits, vegetables, legumes, whole grains, nuts, and seeds, with minimal or no intake of animal products. These diets can vary widely, including vegetarian, vegan, and flexitarian approaches. Despite these variations, all plant-based diets share a common focus on plant-derived foods, which are rich in fiber, antioxidants, phytochemicals, and healthy fats. These nutrients play a crucial role in improving insulin sensitivity, reducing inflammation, and supporting metabolic health [45],[46]. One of the major benefits of plant-based diets is their high fiber content, which helps slow carbohydrate absorption, leading to more gradual rises in blood glucose levels and reducing the overall glycemic load [47]. A number of mechanisms have been put forth to elucidate this phenomenon, including the antioxidant properties of phytochemicals present in plant-based foods and the presence of high concentrations of dietary fiber, which can inhibit glucose absorption, stimulate insulin secretion, reduce hepatic glucose production, and improve intestinal glucose absorption [48],[49]. Furthermore, the diminished prevalence of saturated fatty acids, advanced glycation products, nitrosamines, and heme iron—all of which are linked to insulin resistance and lipotoxicity—can assist in the prevention of insulin resistance and the promotion of insulin sensitivity [50],[51].

#### 2.3.2. Evidence from clinical studies: Effects on glycemic control

Several large cohort studies have shown that plant-based diets are associated with a significantly reduced risk of developing T2D [52]–[54]. The EPIC-Oxford study, which followed over 65,000 participants, revealed that individuals who adhered to a vegetarian diet exhibited a 35% reduction in the risk of developing T2D compared to those who consumed meat. This risk reduction was even more pronounced among vegans, with a 47% reduction in risk [54],[55]. However, these results appear to be attenuated after adjustment for BMI, so the significantly lower risk of developing T2D in vegetarians and vegans appears to be largely or entirely due to lower BMI at baseline [54],[55]. Additional evidence is provided by the Nurses' Health Study (NHS; initiated in 1976) and NHS II (initiated in 1989), two prospective studies that followed approximately 200,000 individuals for a period exceeding 20 years. The findings indicate that greater adherence to a plant-based diet, typified by elevated consumption of fruits, vegetables, whole grains, and legumes and reduced intake of red meat, refined grains, and sugary beverages, was linked to a diminished risk of developing T2D [56]. In contrast, the adoption of a Western dietary pattern, which is typified by higher intakes of red and processed meats, refined cereals, and sugar-sweetened beverages, particularly during adolescence, has been linked to an elevated risk of developing T2D in adulthood [57].

#### 2.3.3. Evidence from longitudinal and meta-analysis studies

A recent meta-analysis of nine prospective cohort studies indicated that high adherence to a plant-based diet was associated with a 23% reduction in the risk of developing T2D compared with low adherence [52]. In conclusion, the current evidence indicates that a plant-based diet may be an effective strategy for the prevention of T2D. Longitudinal studies and meta-analyses indicate that increased adherence to such diets is associated with a notable reduction in the risk of developing the disease. Despite the promising evidence, several areas require further research to fully understand the potential of plant-based diets. These include individual variability, diet composition, comparison with other dietary patterns, duration and long-term adherence, and the development of practical guidelines for implementing plant-based diets in clinical practice, taking into account personal preferences and individual nutritional needs.

### 2.4. High-protein diets

#### 2.4.1. Mechanisms supporting satiety and weight management

High-protein diets (HPDs) are characterized by an increased intake of protein, typically constituting more than 20% of total daily caloric intake, often accompanied by a reduction in carbohydrates or fats. The impact of HPDs on weight management and glycemic control has been extensively studied, yet the results remain inconclusive, with variable efficacy and inconsistent findings [58],[59]. The mechanisms by which increased protein intake may result in a reduction or maintenance of body weight include the following: increased satiety, increased energy expenditure, and improved body composition, which may be achieved by increasing lean muscle mass (LMB) and decreasing fat mass (FM). Specifically, the increase in satiety resulting from protein intake can be attributed to several factors, including increased blood amino acid concentrations, anorexigenic hormones, diet-induced thermogenesis (DIT), and ketone body levels [60].

#### 2.4.2. Conflicting evidence for glycemic control

Several clinical studies have demonstrated the short-term effectiveness of HPDs in improving glycemic control and promoting weight loss. In this context, one study provided evidence that HPDs may be beneficial for weight management, particularly in individuals with prediabetes [61]. Conversely, another study revealed that although HPDs are beneficial for maintaining weight loss in the initial stages, their efficacy diminishes over time due to adherence issues [62]. The relationship between HPDs, T2D prevention, and glycemic control is also controversial [63],[64]. Recent research has indicated that both high- and normal-protein diets are associated with improvements in body composition and glycemic control in patients with T2D; however, high-protein diets have been shown to yield slightly more favorable outcomes [64]. Another paper reports promising results from a study in which 24 obese women and men with prediabetes were recruited and randomized to either an HPD or a high-carbohydrate diet (HCD) for six months. At the conclusion of the study period, the data indicated that the subjects on the HPD had achieved complete remission of prediabetes, whereas only 33% of those on the HC diet had done so, despite similar weight loss. These findings suggest that the HPD, through increased incretin release, may be responsible for improved insulin sensitivity and β-cell function in subjects on the HPD compared to the HCD [63]. A recent review of the literature revealed that HPDs do not significantly enhance glycemic control. However, they may reduce LDL, total cholesterol, triglyceride, and HOMA-IR levels in patients with T2D [65].

#### 2.4.3. Comparison with other dietary approaches

These considerations were revisited in a systematic review and meta-analysis that aimed to compare the effects of different dietary approaches on glycemic control in patients with T2D, thereby highlighting the superiority of the ketogenic diet. In contrast, univariate meta-regressions demonstrated that the mean reduction in HbA1c and FBG were only significantly correlated with the mean change in subjects' weight [66]. Further research is needed to determine whether dietary protein intake plays a role in the prevention or remission of T2D, or whether these effects are primarily due to energy restriction and subsequent weight loss.

### 2.5. The role of gut microbiota in glycemic control and type 2 diabetes

The gut microbiota (GM) significantly influences glycemic control through mechanisms like short-chain fatty acid (SCFA) production, bile acid metabolism, and intestinal barrier regulation. SCFAs, including butyrate, acetate, and propionate, enhance insulin sensitivity by promoting intestinal gluconeogenesis, reducing inflammation, and strengthening gut barrier integrity. Butyrate specifically lowers inflammatory cytokines and lipopolysaccharides (LPS), mitigating insulin resistance [67],[68]. Additionally, SCFAs interact with G protein–coupled receptors (GPR41 and GPR43), stimulating incretin release, including glucagon-like peptide-1 (GLP-1), which enhances insulin secretion [69]. Dysbiosis, or microbial imbalance, is strongly associated with T2D, characterized by reduced butyrate-producing bacteria such as *Faecalibacterium prausnitzii* and *Roseburia*. Prebiotic fibers help restore eubiosis by selectively promoting these beneficial taxa [67],[70]. Bile acid metabolism, influenced by GM, regulates glucose homeostasis via farnesoid X receptor (FXR) and fibroblast growth factor 19 (FGF19) signaling, improving hepatic glucose metabolism [71],[72]. Probiotics containing strains like *Akkermansia muciniphila* and *Lactobacillus* enhance glucose tolerance, improve gut barrier function, and reduce HbA1c levels [73],[74]. Additionally, antidiabetic medications such as metformin appear to exert part of their glucose-lowering effects through alterations in GM composition, further supporting its therapeutic relevance [72]. These findings highlight the potential of GM-targeted interventions—prebiotics, probiotics, and dietary strategies—to improve glycemic control and prevent T2D, though further research is needed to refine and personalize these therapies.

**Figure 2.**
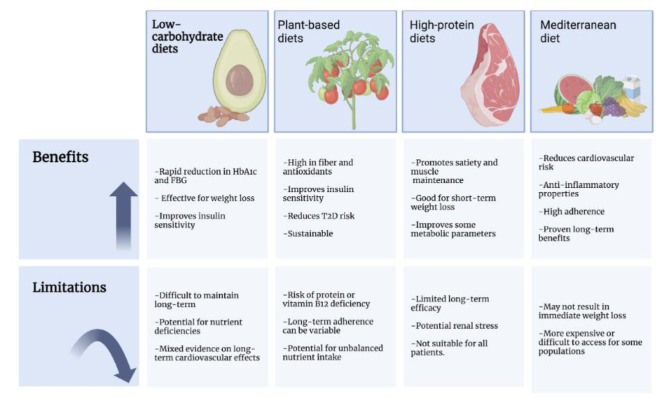
Comparative benefits and limitations of different dietary patterns for type 2 diabetes. This figure illustrates the benefits and limitations of four dietary approaches—Low-Carbohydrate Diets, Plant-Based Diets, High-Protein Diets, and the MD—in the context of T2D prevention. It highlights key outcomes such as weight loss, insulin sensitivity improvement, and cardiovascular health benefits, while also discussing challenges like long-term adherence, nutrient deficiencies, and accessibility.

The comparative benefits and limitations of the primary dietary strategies for T2D—including LCDs, MD, plant-based, and high-protein diets—are summarized in Figure 2.

## 3. Impact of different exercise regimens on type 2 diabetes

### 3.1. Aerobic exercise

#### 3.1.1. Mechanisms and benefits

Fats and carbohydrates are the main sources of energy during exercise, and their relative contribution depends on the duration and intensity of exercise and the type of activity; specifically, endogenous sources include plasma glucose, free fatty acids (FFA) from adipose tissue and lipoproteins, muscle glycogen, and intramyocellular triacylglycerols [75]. Aerobic exercises are activities that engage larger muscles (including activities such as running, jogging, cycling, and swimming) and rely primarily on energy generated by aerobic metabolism [76]. Aerobic training can range in intensity from mild to vigorous and is usually of long duration. Endogenous triacylglycerols represent the body's major energy reserve, most of which is stored in adipose tissue, skeletal muscle, and plasma [77]. During exercise, the FFAs used come mainly from adipose and muscle tissue. Glucose utilization is greatest during high-intensity exercise, while FFA oxidation increases with moderate-intensity exercise [78]. The balance between the metabolic utilization of carbohydrates and lipids during exercise is regulated by the Randle cycle, which inhibits glucose oxidation when fat oxidation is intense and vice versa, with hyperglycemia possibly reducing fat oxidation [79]. It is well known that aerobic activity offers numerous health benefits, helping to prevent or treat a long list of chronic diseases [80]. Among the multitude of parameters improved by aerobic activity, which include but are not limited to improved cardiovascular health, body weight management, and respiratory capacity, is also glycemic control, counteracting the onset or mitigating the complications of T2D [81]–[83].

#### 3.1.2. Evidence from systematic reviews and trials: Impact on blood glucose levels and insulin sensitivity

The effect of aerobic exercise on various aspects of T2D has been extensively studied. A systematic review that analyzed the effects of aerobic exercise training on markers of insulin resistance in overweight or obese children and adolescents found that aerobic exercise was associated with a decrease in fasting insulin levels (−4.52 µU/mL; 95% CI: −7.40 to −1.65; I2, 65%, p = 0.002) and in HOMA (−1.33; 95% CI: −2.47 to −0.18; I2, 73%, p = 0.005) [84]. Furthermore, several studies have demonstrated that in individuals with impaired glucose tolerance (IGT), aerobic exercise, particularly when of high intensity, markedly reduces two-hour postprandial blood glucose levels (2 h-PG) [85],[86]. Short-term aerobic exercise improved insulin sensitivity parameters in adults with T2D and contributed to mitochondrial function [87]. In a study enrolling patients with obesity and T2D, short-term aerobic exercise improved insulin action, specifying that this effect was due to an increase in peripheral insulin sensitivity rather than from hepatic insulin sensitivity [88]. It is also interesting to note that week-long sessions of intense aerobic exercise can improve glycemic parameters without changing body weight, the suggested mechanisms being increased insulin-stimulated glucose disposal and reduced hepatic glucose production [89]. The increased insulin sensitivity induced by aerobic exercise results from an increase in GLUT4 transporter levels in muscle cell membranes by approximately 70% compared with resting levels. This effect occurs in both healthy and insulin-resistant individuals and is attributed to the activation of an independent signaling pathway triggered by muscle contractions [90]. If exercise is prolonged, the increased muscle uptake of glucose remains elevated for approximately 2 h through insulin-independent mechanisms and up to 48 h through insulin-dependent mechanisms [89]. Meta-analyses and systematic reviews confirm that regular aerobic exercise training improves blood glucose in adults with T2D, reducing daily hyperglycemic excursions and lowering HbA1c by 0.5%–0.7% [91],[92]. In addition, regular exercise training improves lipid profile, blood pressure, and fitness levels, even in the absence of weight loss [93],[94].

### 3.2. Resistance training

#### 3.2.1. Benefits for muscle mass, insulin sensitivity, and glucose uptake

It is well established that indices of skeletal muscle function serve as an indicator and, in certain instances, may even act as a determinant of overall health [95]. A weak grip has been linked to an elevated risk of mortality from all causes and cardiovascular disease. Conversely, a strong grip has been demonstrated to reduce the risk of mortality from all causes and cardiovascular disease in individuals with T2D [96],[97]. During the fasting state, skeletal muscle serves as the primary protein reserve in the body, playing a pivotal role in replenishing circulating amino acids derived from tissues such as the heart, brain, liver, and skin to sustain protein synthesis [98]. The significance of this topic is underscored in circumstances where there is a reduction in muscle mass, such as in sarcopenia. This is an age-related condition that is characterized by a decline in muscle mass and functionality, which in turn increases the risk of falls, disability, and a reduction in quality of life [99]. Furthermore, the loss of skeletal muscle mass is accelerated in several disease states, including T2D [100]. A study involving 485 participants revealed significant differences in skeletal muscle mass and function between individuals with and without T2D. The findings indicated that individuals with T2D exhibited reduced muscle strength values of 3%–6% and reduced muscle quality values of 7%–8% [100]. In individuals with T2D, skeletal muscle serves as the primary site for glucose management, with approximately 80% of postprandial glucose being absorbed in muscle tissue. However, insulin resistance in muscle tissue is regarded as a significant defect in the pathogenesis of T2D [101]. Notwithstanding the maintenance of muscle mass, the uptake of glucose by muscles is approximately 60% lower in individuals with T2D mellitus (T2DM). This suggests that other factors, such as muscle strength and quality, are of paramount importance for the sustenance of metabolic health [102]. In this regard, resistance exercise, also known as strength training, plays a crucial role in maintaining and improving muscle mass and function [103],[104]. This type of exercise entails the utilization of free weights, strength training apparatus, or resistance bands for the purpose of stimulating muscle growth and enhancing strength.

#### 3.2.2. Evidence from RCTs on fasting glucose and HbA1c

The results of numerous clinical studies have prompted a discussion regarding the efficacy of resistance training in managing glycemic irregularities. Some research has indicated that, despite the benefits of strength exercises, the use of elastic bands does not result in significant improvements in HbA1c levels [105]. Nevertheless, other research has yielded disparate findings. Recent studies have underscored the importance and necessity of a systematic resistance exercise program for the effective management of insulin levels, blood sugar levels, and other cardiovascular risk factors [106]. The results of several clinical trials in patients with prediabetes and T2D have demonstrated that resistance training is an effective method for reducing FBG levels [107]. These findings are supported by a recent meta-analysis that identified low-to-moderate resistance training as the best intervention for improving FBG [108]. Individuals with T2D who participated in the training program exhibited improvements in strength, skeletal muscle mass, insulin sensitivity, and other cardiometabolic factors, with an average increase of up to 15% [109]. In particular, in the context of a negative caloric balance and modest weight loss, resistance training has been demonstrated to enhance lean muscle mass and diminish HbA1c levels to a degree that is three times more pronounced in older adults with T2D than in those who engage in a low-calorie diet in the absence of exercise, resulting in a loss of muscle mass [110]. A meta-analysis on resistance exercise indicates that high-intensity training is more effective than low- or moderate-intensity training for overall glucose management and reduction of insulin levels in adults with T2D. This underscores the importance of intensity as the primary determinant of the efficacy of resistance exercise, particularly in relation to the fitness level of the individual [106].

#### 3.2.3. Challenges in adoption and adherence

Adopting and adhering to resistance training for T2D can be challenging due to misconceptions about equipment needs, physical demands, and injury risks, particularly for beginners or older adults. Time constraints, low motivation, and lack of knowledge about proper techniques further hinder consistency. Addressing these barriers through education, supervised programs, and accessible, home-based options can improve adherence and long-term benefits [111].

### 3.3. High-intensity interval training (HIIT)

#### 3.3.1. Efficiency and outcomes

High-intensity interval training (HIIT) has emerged as one of the most popular exercise programs in recent years. HIIT consists of short bursts of vigorous activity (about 90% of maximum aerobic power for short intervals), followed by short periods of rest or low-intensity activity for recovery [112]. Because of the very high exercise intensity, HIIT may present a higher risk of injury; nevertheless, health benefits occur when implemented correctly [113]. In addition, the ability of this exercise protocol to induce significant physiological and metabolic adaptations, together with its short duration in terms of time, make it an appropriate option to overcome the “lack of time” that characterizes today's society, which is often the most common obstacle to physical activity [114]. Studies on HIIT that have employed treadmills or cycling on ergometers suggest that this method of physical activity can be a viable alternative to continuous moderate-intensity exercise (MICT) or resistance exercise to promote various health benefits, such as improving aerobic capacity, lipid metabolism, vascular function, and glycemic control [115],[116].

#### 3.3.2. Effects on glycemic control, weight management, and cardiovascular health

It is interesting to note that a single HIIT session consisting of ten 1-min intervals at 90% of maximum heart rate improves postprandial glycemic control for 24 h in subjects with T2D, compared with controls who did not exercise [117]. In addition to this, compared with MICT of 30 min at 65% of maximum heart rate, the same HIIT protocol is equally effective in reducing postprandial hyperglycemia on the day of exercise, with an improvement in muscle insulin sensitivity until the day after training [118]. Recent studies have associated acute HIIT with significant improvements in postprandial, overnight, and FBG concentrations compared with MICT with equivalent exercise volume [119]. A meta-analysis that analyzed the effects of HIIT on markers of glucose regulation and insulin resistance compared with control conditions (CON) or continuous training (CT) revealed a reduction in insulin resistance following HIIT compared with both CON and CT [HIIT vs. CON: standardized mean difference (SMD) = −0.49, CIs: −0.87 to −0.12, p = 0.009; CT: SMD = −0.35, −0.68 to −0.02, p = 0.036). Compared with CON, HbA1c decreased by 0.19% (−0.36 to −0.03, p = 0.021) and body weight decreased by 1.3 kg (−1.9 to −0.7, p < 0.001). Specifically, participants at risk of T2D experienced reductions in FBG (−0.92 mmol/L, −1.22 to −0.62, p < 0.001) compared with CON [120]. An additional aspect to consider during a HIIT exercise session is the volume of exercise performed. In this regard, a randomized, controlled trial conducted to compare the effect of low-volume HIIT (LV-HIIT) with high-volume HIIT (HV-HIIT) on HbA1C and FBG in overweight adults with prediabetes showed that both exercise interventions had statistically significant effects on HbA1C and FBG (p < 0.05) compared with controls; however, HV-HIIT produced greater reductions in HbA1C than LV-HIIT (26.07% vs. 14.50%) and FBG (17.80% vs. 13.22%) after exercise, being more effective in counteracting progression to T2D in subjects with prediabetes [121].

#### 3.3.3. Cautionary notes for T2D patients

HIIT, therefore, appears to be an effective method for improving metabolic health and could be a time-efficient option for patients with T2D who can tolerate it. However, people with T2D should closely monitor their responses to training, as chronic intense training may cause transient post-exercise hyperglycemia [122]. Based on these considerations, it can be inferred that although HIIT is as effective as MICT in reducing postprandial hyperglycemia, further research is needed to determine the candidacy of patients to perform this protocol and the minimum effective dose of HIIT to improve glycemic control along with the maximum tolerable dose to avert metabolic complications related to glucose homeostasis in patients with T2D.

### 3.4. Combined exercise programs

#### 3.4.1. Synergistic effects of aerobic and resistance training

The combination of aerobic and resistance training has been extensively studied, demonstrating significant benefits for T2D prevention and management. Recommendations from organizations such as the WHO, ACSM, CDC, and ADA strongly support this approach for enhancing glycemic control and lipid profiles in individuals with metabolic disorders [123]. The combined use of different types of exercise has been demonstrated to result in an improved glycemic profile. This is achieved through a range of mechanisms, including improvements in anthropometric and cardiometabolic parameters. These improvements include increased muscle mass and function, as well as a reduction in ectopic fat. This is due to an enhanced ability to mobilize lipids and oxidize fatty acids in the mitochondria [124]. The evidence for this assertion is drawn from a recent meta-analysis of 24 studies involving 1946 patients with prediabetes and 7 exercise intervention models. The analysis demonstrated that changes in glycemic levels combining moderate-intensity aerobic exercise with low- or moderate-load resistance training yielded the most significant improvements in HbA1c, BMI, body weight, total cholesterol (TC), and low-density lipoprotein cholesterol (LDL). These findings support the efficacy of this intervention as a strategy for preventing T2D [108]. It is noteworthy that when resistance exercise is conducted prior to aerobic exercise within the same session, as opposed to as a standalone session, glucose levels remain more stable and instances of hypoglycemia are less frequent, both during and following the completion of the training session [125]. A study of 28 postmenopausal women with T2D demonstrated that 16 weeks of combined exercise training resulted in enhanced insulin sensitivity, attributable to augmented insulin-mediated glucose uptake, in the cohort undergoing combined exercise rather than in the group engaged in isolated aerobic exercise [126]. Another study assessed the impact of a year-long combined exercise regimen on 120 patients with T2D. The findings indicated a notable improvement in HbA1c levels (8.31% ± 1.73% to 7.1% ± 1.16%, p < 0.001), as well as a reduction in FBG (165 ± 60.6 to 129 ± 37 mg/dL, p < 0.0001) compared with the control group. These results support the hypothesis that the combined exercise approach may offer additional benefits through a synergistic action of the two types of exercise [127]. A meta-analysis of 27 studies that analyzed the effects of different exercise modalities on measures of glucose control and other risk factors for T2D complications showed that all three exercise modalities have a positive impact on blood glucose and insulin sensitivity; however, combined exercise results in greater reductions in HbA1c than either of the isolated exercise modalities [128]. These findings were corroborated by another systematic review of 14 randomized controlled trials, which concluded that combined exercise represents the most efficacious strategy for improving glycemic control and lipid profile in patients with T2D [129].

**Figure 3.**
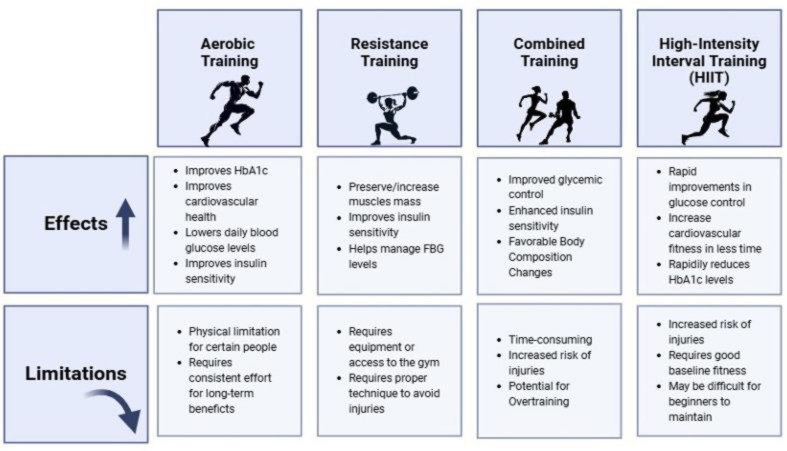
Comparative effects and limitations of different exercise regimens on type 2 diabetes. This figure summarizes the effects and limitations of three major types of exercise—Aerobic Exercise, Resistance Training, Combined Training and High-Intensity Interval Training (HIIT)—on T2D management. The chart outlines key benefits such as improvements in HbA1c, insulin sensitivity, cardiovascular health, and glucose control for each exercise type. Limitations of each regimen are also presented, including factors like time commitment, injury risk, and the need for equipment or specific techniques.

A visual comparison of the benefits and limitations of aerobic exercise, resistance training, and HIIT in the context of T2D management is presented in Figure 3.

#### 3.4.2. Practical recommendations

In conclusion, it can be posited that a combined exercise approach may prove to be the optimal strategy for glycemic management in patients with T2D when compared with aerobic or resistance exercise alone. Further research should consider analyzing and quantifying the additive effects resulting from the synergistic action of this type of exercise in comparison with its individual component exercise modalities.

## 4. Discussion

The global T2D epidemic reflects profound social and economic changes. Urbanization and technological advancements have replaced physically demanding jobs with sedentary lifestyles [130]. Concurrently, economic growth has reshaped food systems, increasing the availability of calorie-dense processed foods and reducing access to fresh produce. These dietary shifts, characterized by high intakes of processed meats, refined carbohydrates, and sugary drinks, are closely linked to rising rates of obesity and T2D [130],[131]. Projections indicate that by 2030, prediabetes will affect 40% of the U.S. population, doubling its prevalence from 2000 [132]. This trend may be mitigated by weight loss resulting from lifestyle modifications during the prediabetic state [133]. In this regard, large-scale randomized clinical trials, such as the Diabetes Prevention Program (DPP) and the Finnish Diabetes Prevention Study (FDPS), have demonstrated, with unequivocal evidence, that weight loss achieved through a careful combination of a low-calorie diet and regular exercise (150–210 min per week) can reduce the risk of developing T2D in people with prediabetes by up to 50% [134],[135]. These studies have involved thousands of participants and have demonstrated that even a modest weight loss of 5%–7% of initial body weight is sufficient to achieve significant benefits [134],[135]. Although the amount of weight loss emerges as the determining factor in the prevention of T2D, this review also aims to evaluate the role of diet beyond weight loss.

### 4.1. Additional evidence supporting Mediterranean and plant-based diets

Different dietary regimens, due to different macronutrient compositions and levels of caloric restrictions, could have different effects on the mechanisms regulating glucose homeostasis, even when weight loss is equivalent [136],[137]. Therefore, other diet- or lifestyle-related factors, not necessarily related to the absolute amount of weight loss, are likely to be involved in improving the control of glucose homeostasis and the prevention of T2D. Of these, the MD and plant-based diets have provided the most consistent evidence, suggesting that elements common to both dietary patterns, such as an abundant intake of fruits, vegetables, whole grains, legumes, and nuts, may contribute to improved glycemic homeostasis [42],[52]. Of particular relevance is the inverse association between the degree of adherence to the MD and the risk of developing T2D [37].

Additionally, plant-based diets, advocating for reduced consumption of animal-derived foods, demonstrate substantial benefits in lowering glycemic indices and reducing cardiovascular markers, making them attractive strategies for T2D prevention [138]. Notably, objective dietary biomarkers have begun to validate these findings and address the inherent limitations of self-reported adherence [139].

Other dietary approaches such as LCDs may be effective in improving glycemic control and reducing cardiovascular risk in patients with prediabetes or overt T2D by significantly reducing HbA1c, plasma glucose, and body weight levels [30]. However, long-term benefits are limited by the difficulty of adherence to this type of diet [33]. Finally, although HPDs have been shown to reduce levels of LDL, total cholesterol, triglycerides, and HOMA-IR in patients with T2D, they show variable effects on weight management and glycemic control, leading to inconsistent results on their usefulness in preventing T2D [58],[65]. Proposed hypotheses that different dietary regimens could positively influence glucose homeostasis extend beyond the simple mechanism of weight loss; they include the modulation of inflammation, the improvement of insulin sensitivity, and the regulation of the gut microbiota [140],[141]. These findings offer a spectrum of nutritional alternatives for the prevention of T2D, with a focus on comprehensive dietary quality, treatment objectives, and individual and cultural culinary preferences.

### 4.2. Emerging role of digital and structured lifestyle interventions

Lifestyle modifications, including structured diet and exercise programs guided by specialized dietitians, have shown significant benefits. These interventions, conducted in ambulatory settings, reduce carbohydrate intake, improve anthropometric measures, and promote regular physical activity [142]. However, digital health technologies are emerging as valuable tools to overcome barriers such as poor adherence to traditional exercise programs. Tools like digital voice assistants can facilitate patient-practitioner communication and support self-management, particularly among older adults and underserved populations [143].

The synergistic effects of a Mediterranean diet combined with aerobic and anaerobic exercises have been examined in the CPNET study, which evaluated the impact of a Mediterranean-type dietary pattern supplemented with whey protein and regular physical activity (50% aerobic exercise and 50% resistance training) on obese individuals with prediabetes and T2DM. The results of the study indicated a significant reduction in fasting plasma glucose levels among the prediabetic participants, along with a modest improvement in HbA1c levels in the T2D group [144].

A plant-based diet in conjunction with physical activity has been demonstrated to be an effective treatment for T2D. This is due to the fact that a plant-based diet is rich in fiber and low in saturated fats, which improves insulin sensitivity. Additionally, plant-based diets tend to be lower in calories compared to other diets, thereby assisting individuals with T2D in the management of their weight [145].

### 4.3. The role of exercise in T2D management

Regular physical activity is also widely recognized as an essential component in the primary and secondary prevention of T2D, contributing to weight control and improving insulin sensitivity, blood pressure, and lipid profile [146]. In this regard, the Chinese Da Qing study demonstrated that increased physical activity, even in the absence of significant weight loss, can contribute to the reduction of the risk of developing T2D [147]. It is also noteworthy that the beneficial effects of lifestyle modifications are not merely transient; a number of studies have demonstrated that the benefits of weight loss and increased physical activity can persist for many years, even more than a decade after the active intervention has ceased [148]. The results of this review identified aerobic exercise as the best intervention to improve 2h-PG and blood pressure. Several clinical studies support this evidence by showing that aerobic exercise reduces 2h-PG levels and improves diastolic and systolic blood pressure parameters in patients with T2D and those with prediabetes [85],[86],[149]. Effects on blood pressure would result from increased synthesis and action of nitric oxide, a potent vasodilator, and improved endothelial function [150]. Increased peak oxygen consumption resulting from aerobic activity exerts a beneficial influence on multiple facets of metabolic well-being, extending beyond glucose tolerance. This is particularly evident in the enhancement of systemic insulin sensitivity, reduction of daily hyperglycemic fluctuations, and lowering of HbA1c [87],[91],[92]. Additionally, aerobic exercise has been shown to improve lipid profile and fitness levels [93],[94]. In contrast to strength training, aerobic exercise effectively stimulates the expression of GLUT4, facilitating glucose uptake into muscle cells and thus helping to regulate blood sugar levels [90]. While there is currently no definitive evidence that alteration of pancreatic beta cell function is directly related to maximal aerobic capacity (VO2max), some research indicates that exercise of moderate and vigorous intensity may improve the function of these cells through several mechanisms, including increasing insulin sensitivity and compensatory reduction of insulin secretion [150],[151]. Aerobic exercise, generally categorized as MICT or HIIT, offers numerous health benefits [118]. In particular, HIIT has been shown to significantly improve aerobic capacity, lipid metabolism, and glycemic control [115],[116]. Clinical studies have associated acute HIIT sessions with significant improvements in postprandial, overnight, and FBG compared to MICT, even for the same exercise volume [119]. This benefit may be due to the lower production of reactive oxygen species and the resulting lower oxidative stress induced by HIIT compared with MICT [152]. This allows for better preservation of endothelial function and increased bioavailability of nitric oxide [152]. The combination of effectiveness and practicality makes this protocol an ideal choice for overcoming time constraints and integrating physical activity into daily life. However, further studies are needed to determine the training modalities and candidacy of patients to perform this protocol to avoid metabolic complications in patients with impaired glucose homeostasis.

### 4.4. Integrating aerobic and resistance training

In contrast to aerobic exercise, resistance training was identified as the most effective form of exercise for improving FBG [108]. The biological mechanisms by which resistance training induces a series of cellular adaptations that improve insulin sensitivity and reduce FBG include the accumulation of glycogen through the activation of glycogen synthase and the stimulation of muscle protein synthesis by the activation of the IGF-1/PI3K/AKT system [153],[154]. Furthermore, transient activation of AMPK facilitates the translocation of the glucose transporter GLUT-4, thereby increasing glucose uptake and enhancing fatty acid oxidation in skeletal muscle [155]. Although some studies have indicated a potential association between augmented muscle mass and enhanced glycemic regulation, other research has not corroborated this hypothesis [105]. This discrepancy may be attributed to the fact that muscle function, rather than muscle mass alone, plays a pivotal role in glycemic control [156]. Nevertheless, the scientific literature on this topic remains controversial and requires further investigation. The intricate nature of the underlying mechanisms and the considerable inter-individual variability render it challenging to establish a straightforward, causal relationship. The paucity of studies on particular subgroups of patients with prediabetes, coupled with the heterogeneity of training regimens employed, precludes the possibility of extrapolating the findings to a broader population. The combined effects of aerobic and resistance training have been extensively studied [157],[158]. Such evidence suggests that a combination of moderate-intensity aerobic exercise and low- to moderate-intensity resistance training can improve HbA1c, body weight, and total and LDL cholesterol, mitigating cardiovascular risk and reducing the risk of developing T2D [108]. Recommendations to perform a combination of aerobic and resistance training come from prestigious institutions, including the WHO, American College of Sports Medicine (ACSM), Centers for Disease Control and Prevention (CDC), and current ADA guidelines to improve glycemic control and lipid profile in patients with metabolic disorders [17],[123],[159],[160]. Our analyses support the use of combined exercise as an effective strategy for the prevention of T2D and cardiovascular disease. This approach works by multiple mechanisms: it stimulates the AKT/PKB-associated insulin signaling pathway, increasing glucose uptake at the cellular level [161]; it enhances GLUT4 expression, promoting glucose utilization by muscle and improving insulin resistance [161],[162]; and finally, it modulates the release of adipokines, including leptin and adiponectin, promoting fatty acid oxidation, reducing visceral fat and vascular lipid deposition, positively impacting cardiovascular risk [163]. Based on this evidence, and considering the difficulties obese and overweight individuals have in performing physical activity, moderate-intensity aerobic exercise combined with low-to-moderate-intensity resistance exercise is strongly recommended for managing glucose metabolism and preventing T2D.

### 4.5. Dietary insights on meat consumption and intermittent fasting

Finally, dietary guidelines should address the role of red and processed meats as significant risk factors for T2D development. A meta-analysis across diverse populations highlights the need to reduce meat consumption to mitigate this risk [164]. Additionally, intermittent fasting protocols, which restrict eating to specific time windows, offer promising alternatives for weight loss and glycemic control, independent of continuous energy restriction [138]. These strategies, tailored to individual preferences and cultural factors, provide practical options for improving adherence and preventing T2D. A recent review investigated various intermittent fasting approaches for individuals with diabetes, including alternate-day fasting (wherein subjects alternate between fasting and normal eating days), the 5:2 diet (wherein subjects consume a normal diet for five days a week and consume 500–600 calories on two non-consecutive days), and time-restricted feeding (wherein subjects consume all meals within a specific time window followed by a fasting period). The findings of the review indicated that intermittent fasting can improve various health parameters, including, but not limited to, insulin sensitivity, weight management, and cardiovascular health [165].

## 5. Recommendations

### 5.1. For individuals

Adopting a healthy lifestyle is essential for preventing and managing T2D. Individuals should prioritize dietary patterns such as the MD and plant-based diets, both of which have demonstrated substantial benefits for glycemic control, insulin sensitivity, and cardiovascular health. These diets emphasize fruits, vegetables, whole grains, legumes, nuts, and healthy fats while limiting processed foods, refined carbohydrates, and saturated fats. Reducing red and processed meat consumption is particularly beneficial for lowering T2D risk. Additionally, intermittent fasting, which promotes glycemic control independent of weight loss, offers a flexible and effective dietary approach. Physical activity should be an integral part of lifestyle changes, with a focus on at least 150 min of moderate-intensity aerobic exercise combined with resistance training 2–3 times per week. HIIT is a time-efficient alternative for those with limited availability, providing significant improvements in glycemic control, lipid metabolism, and aerobic capacity. Digital tools such as mobile apps or voice assistants can support adherence by integrating exercise into daily routines and addressing common barriers.

### 5.2. For healthcare providers

Healthcare providers should play an active role in guiding patients toward sustainable lifestyle changes. Recommending evidence-based dietary interventions such as the MD and plant-based diets can help patients achieve better glycemic control and prevent T2D complications. Dietary advice should be tailored to cultural preferences, socioeconomic factors, and individual goals to maximize adherence. Programs involving dietitians and structured meal planning have proven successful and should be emphasized in both primary and specialized care settings. For physical activity, a combination of aerobic and resistance training remains the cornerstone of T2D prevention and management. HIIT should also be discussed as an alternative for time-constrained patients. Addressing concerns such as hypoglycemia fears or physical limitations and recommending accessible solutions like digital health tools can further enhance adherence and outcomes.

### 5.3. For future research

Future research should focus on personalizing lifestyle interventions by exploring the interplay of diet, exercise, and emerging areas such as the gut microbiota. Investigating individual variability in metabolic responses to dietary and exercise regimens will provide deeper insights into tailoring interventions. The role of digital health technologies in delivering scalable, cost-effective lifestyle programs also warrants further exploration. Long-term, real-world studies are needed to evaluate the sustainability and impact of these integrative approaches across diverse populations.

## 6. Conclusions

This review underscores the critical role of lifestyle interventions, particularly dietary patterns and physical activity, in the prevention and management of T2D. Evidence highlights the effectiveness of Mediterranean and plant-based diets, alongside combined aerobic and resistance exercise, in improving glycemic control, insulin sensitivity, and cardiovascular health. However, the variability in individual responses to these interventions necessitates a more personalized approach to maximize their effectiveness. Future research should focus on determining the most effective combinations of dietary and exercise strategies tailored to the specific characteristics of each patient. This includes considering individual metabolic profiles, preferences, and capabilities to ensure adherence and sustainability. Importantly, this approach should also account for the different trajectories in the development of T2D and the recognized subtypes of the disease. Tailored interventions must address the heterogeneity in disease progression, risk factors, and patient goals. To advance this personalized approach, further investigation is needed into emerging fields such as the role of gut microbiota, genetic and metabolic profiling, and behavioral factors that influence adherence. Longitudinal studies and real-world implementation research are essential to develop scalable, practical solutions that integrate personalized strategies into diverse clinical and public health settings. By addressing these challenges, the burden of T2D can be significantly reduced, offering improved outcomes for individuals and healthcare systems globally.
